# Safety and pharmacokinetics of single and multiple doses of ledaborbactam etzadroxil with or without ceftibuten in healthy volunteers

**DOI:** 10.1128/aac.00210-25

**Published:** 2025-08-05

**Authors:** Carlos Fernando de Oliveira, Mary Beth Dorr, Jeroen van de Wetering, Kathryn Lowe, Philip Sabato, Gregory Winchell, Hongzi Chen, Paul C. McGovern

**Affiliations:** 1 Venatorx Pharmaceuticals, Inc., Malvern, Pennsylvania, USA; 2 PRA Health Sciences, Groningen, the Netherlands; 3 Winchell Pharma Consulting, LLC 509759 , Norristown, Pennsylvania, USA; University of California San Francisco, San Francisco, California, USA

**Keywords:** ceftibuten, ledaborbactam, ledaborbactam etzadroxil, VNRX-7145, VNRX-5236, beta-lactamase inhibitor, drug safety, pharmacokinetics, first-in-human

## Abstract

Ledaborbactam etzadroxil (LED-E), a novel oral prodrug that converts to the active β-lactamase inhibitor (BLI) ledaborbactam (LED), is being developed in combination with ceftibuten (CTB) to address a need for oral treatments against drug-resistant Enterobacterales infections. Two Phase 1 studies were conducted to (i) evaluate the safety and pharmacokinetics of single doses of LED-E 100 to 1000 mg and multiple doses of LED-E 75 to 500 mg every 8 h (q8h) for 10 days, (ii) assess potential drug interactions between CTB and LED, and (iii) evaluate safety and pharmacokinetics following multiple doses of LED-E 300 or 500 mg with CTB 400 mg or placebo q8h for 10 days. Treatment-emergent adverse events (TEAEs) were reported for 82% LED-E ± CTB (*n* = 109) and 78% placebo (*n*=27) participants, respectively. TEAEs of gastrointestinal disorders were reported for 28% of the LED-E ± CTB and 15% of placebo participants. Following single doses, LED-E AUC_inf_ was less than 2% of LED exposures, demonstrating extensive conversion to active BLI. LED AUC increased dose proportionally following single LED doses and less than proportionally following multiple LED-E doses. After 10 days of q8h dosing, the LED terminal half-life in plasma was approximately 11 to 12 h. Steady-state urinary excretion of LED-E–derived material (comprised almost entirely of unchanged LED) was 84%. No clinically relevant CTB and LED PK interactions occurred with the CTB + LED-E combination. These results support further development of the CTB + LED-E combination for the treatment of complicated urinary tract infections caused by drug-resistant Enterobacterales.

**CLINICAL TRIALS:**

These studies are registered with ClinicalTrials.gov as NCT04243863 and NCT04877379.

## INTRODUCTION

Drug-resistant Gram-negative pathogens pose a critical public health threat (1, 2). Resistance has been demonstrated to nearly all antibiotic classes, including last-resort options, such as carbapenems and colistin (3). Rapid spread and acquisition of resistance genes leave fewer effective treatments, straining healthcare systems and increasing morbidity and mortality (3, 4). This underscores the urgent need for new antibiotics to treat infections due to antimicrobial-resistant pathogens (1, 2).

Ledaborbactam etzadroxil (LED-E; formerly VNRX-7145) is an oral prodrug of ledaborbactam (LED; VNRX-5236), a novel broad-spectrum β-lactamase inhibitor. Ceftibuten (CTB; Cedax), a broad-spectrum, third-generation oral cephalosporin, and LED-E represents a new β-lactam/β-lactamase inhibitor combination designed to address the urgent need for oral treatments effective against drug-resistant Gram-negative pathogens. CTB + LED-E restores CTB activity against Enterobacterales expressing challenging resistance mechanisms, including extended-spectrum β-lactamases (ESBLs) and carbapenemases, such as KPC and OXA-48 (3).

Here, we describe results from two Phase 1 studies from the CTB + LED-E drug development program. The first-in-human study VNRX-7145-101 (NCT04243863) focused on the safety and pharmacokinetics following single and multiple doses of LED-E in healthy participants. VNRX-7145-102 (NCT04877379) expanded on this by evaluating the potential for drug-drug interactions between CTB and LED and the safety and pharmacokinetics of multiple doses of the CTB + LED-E combination in healthy participants.

## RESULTS

### Disposition and participant demographics

Across both studies, 136 participants (LED-E ± CTB, *n* = 109; placebo, *n* = 27) were enrolled and received at least one dose of study drug, and 132 participants completed all study procedures. One participant in the LED-E 150 mg every 8 h (q8h) group discontinued due to a TEAE; all other discontinuations were due to loss to follow-up or protocol noncompliance.

Table S1 summarizes demographics and clinical characteristics at baseline for all participants. The mean age was 31.0 years (SD, 8.5 years; range, 18 to 53 years), 56.6% of participants were female, 70.6% were White, 17.6% were Black or African American, and 40.4% were Hispanic or Latino. The distribution of these characteristics was similar between the pooled LED-E ± CTB and placebo groups, except for the percentage of White patients, which was lower in the placebo group (59.3%) than in the CTB + LED-E (73.4%) group. The mean body mass index was 25.5 kg/m^2^ (SD, 3.2 kg/m^2^; range, 18.9 to 31.9 kg/m^2^).

### Safety and tolerability

The numbers of participants with treatment-emergent AEs (TEAE) were similar in the LED-E ± CTB (81.7%) and placebo (77.8%) groups (Table 1). Among LED-E-dosed participants, headache (20.2%) and fatigue (9.2%) were the most frequently reported TEAEs. Gastrointestinal TEAEs occurred in 27.5% of LED-E-dosed participants and 14.8% of placebo-dosed participants, were all mild in severity, and did not lead to discontinuation of study drug. There was no dose relationship with the frequency or severity of TEAEs for gastrointestinal events.

**TABLE 1:** Treatment-emergent adverse events in ≥2% of healthy participants who received ledaborbactam etzadroxil (LED-E) with or without ceftibuten (CTB) or placebo^*a*^

TEAE^*b*^	LED-E ± CTB^*c*^ (*n* = 109)	Placebo (*n* = 27)	Total (*N* = 136)
Any TEAE	89 (81.7)	21 (77.8)	110 (80.9)
Gastrointestinal disorders	30 (27.5)	4 (14.8)	34 (25.0)
Abdominal pain	9 (8.3)	2 (7.4)	11 (8.1)
Constipation	4 (3.7)	0	4 (2.9)
Frequent bowel movements	7 (6.4)	0	7 (5.1)
Nausea	9 (8.3)	0	9 (6.6)
General disorders and administration site conditions	23 (21.1)	5 (18.5)	28 (20.6)
Fatigue	10 (9.2)	1 (3.7)	11 (8.1)
Nervous system disorders	25 (22.9)	4 (14.8)	29 (21.3)
Dizziness	5 (4.6)	1 (3.7)	6 (4.4)
Headache	22 (20.2)	2 (7.4)	24 (17.6)
Somnolence	4 (3.7)	0	4 (2.9)

^*a*^ Data include all participants from studies VNRX-7145-101 and VNRX-7145-102 who received ≥1 dose of study medication.

^*b*^ TEAE, treatment-emergent adverse event. TEAEs were defined as adverse events with onset date-times on or after the date-times of first exposure to study treatments.

^*c*^ bCTB, ceftibuten; LED-E, ledaborbactam etzadroxil.

One participant in the LED-E 150 mg q8h group discontinued the study due to a mild, related TEAE of noncardiac chest pain. No serious adverse events or deaths occurred. No clinically relevant changes were noted in clinical laboratory values, ECGs, or vital signs.

### Pharmacokinetics

#### Single doses of LED-E

##### Plasma results

**Fig 1:**
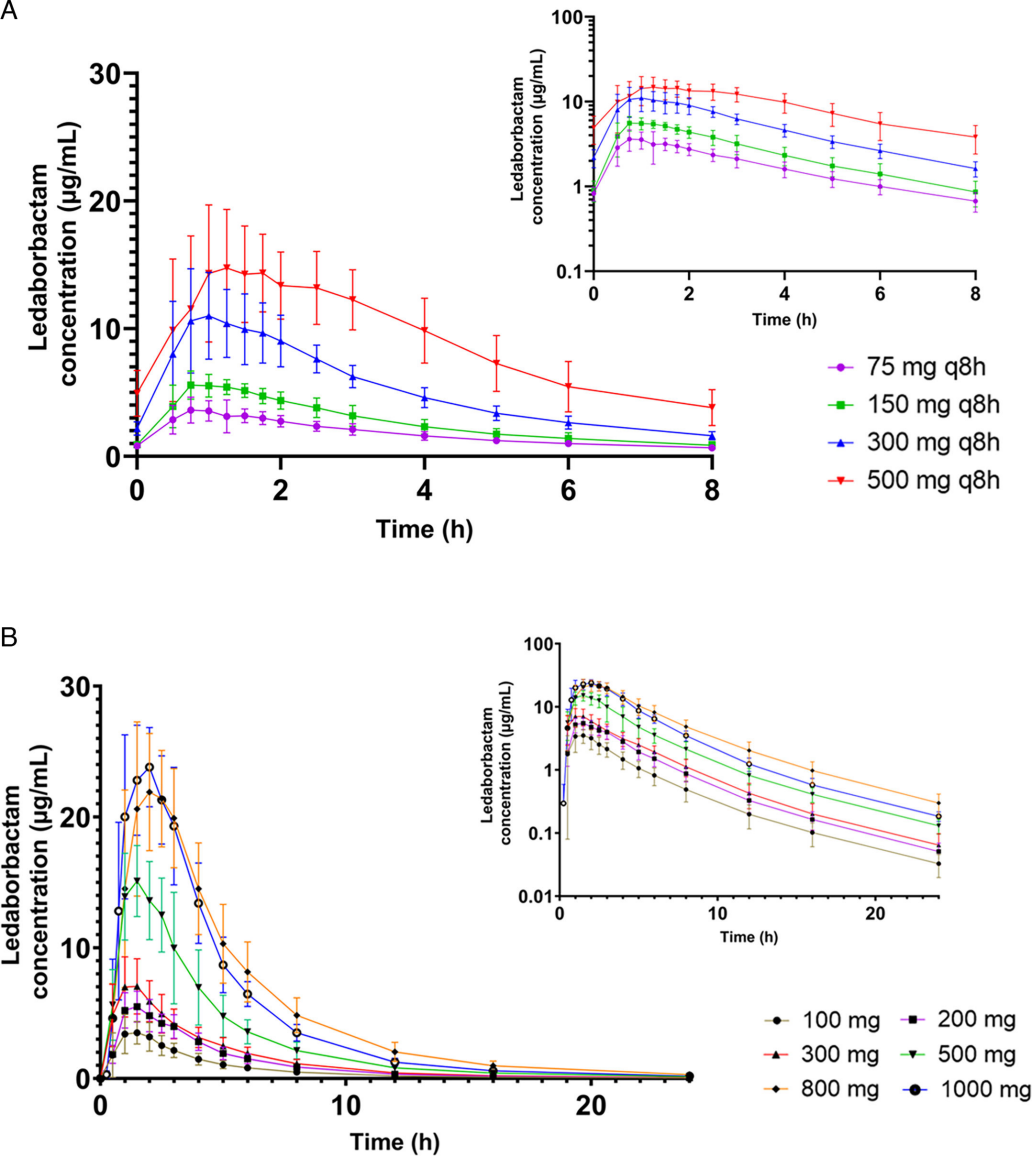
Arithmetic mean plasma ledaborbactam concentrations following (**A**) single doses and (**B**) multiple doses (Day 10) of ledaborbactam etzadroxil. Primary figures show linear scale; insets show logarithmic scale.

Pharmacokinetic parameters were assessed following a single dose of LED-E 100 to 1,000 mg. Arithmetic mean plasma concentration-time profiles for LED are shown in Fig. 1A. LED concentrations increased rapidly after administration, reaching *C*_max_ at 1.25 to 2 h post-dose, then decreased in a biphasic manner over time. LED exposure generally increased dose proportionally, with geometric mean AUC_inf_ values of 15.400 h·µg/mL (LED-E 100 mg) to 114.000 h·µg/mL (LED-E 1000 mg). LED *C*_max_ and AUC_inf_ exhibited low variability across the dose range (Table 2).

**TABLE 2:** Plasma pharmacokinetic parameters for ledaborbactam and ledaborbactam etzadroxil, following single doses of ledaborbactam etzadroxil (LED-E)^*a*^

Parameter^*b*^	LED-E 100 mg (*n* = 6)	LED-E 200 mg (*n* = 6)	LED-E 300 mg (*n* = 6)	LED-E 500 mg (*n* = 6)	LED-E 800 mg (*n* = 6)	LED-E 1,000 mg (*n* = 6)
Ledaborbactam						
*T*_max_, h^*c*^	1.25 (0.50–2.00)	1.50 (1.00–3.00)	1.25 (0.50–2.00)	1.26 (1.00–3.00)	2.00 (1.00–3.00)	2.00 (1.00–3.00)
*C*_max_, µg/mL	4.010 (25.6)	5.970 (11.8)	7.570 (29.3)	16.800 (8.43)	24.000 (14.2)	26.500 (7.92)
AUC_inf_, h·µg/mL	15.400 (30.5)	26.200 (16.7)	33.100 (25.6)	69.300 (15.2)	125.000 (22.6)	114.000 (8.82)
*t*_1/2_, h	5.53 (18.7)	6.10 (27.4)	7.48 (32.4)	11.3 (34.5)	8.59 (42.9)	11.3 (19.1)
Ledaborbactam etzadroxil						
*T*_max_, h^*c*^	0.50 (0.50–1.00)	0.50 (0.50–1.00)	0.50 (0.50–1.50)	0.51 (0.50–2.50)	0.50 (0.50–1.50)	0.75 (0.50–0.75)
*C*_max_, µg/mL	0.167 (79.7)	0.167 (57.6)	0.347 (95.5)	0.917 (112)	1.160 (95.3)	1.840 (21.2)
AUC_inf_, h·µg/mL	0.177 (53.4)	0.249 (23.3)	0.439 (52.7)	1.290 (67.4)	1.720 (68.0)	2.300 (11.2)
*t*_1/2_, h	2.81 (24.5)	3.34 (6.35)	3.63 (17.3)	4.70 (13.3)	3.37 (14.2)	4.59 (7.73)
CL/F, L/h	564 (53.4)	803 (23.3)	683 (52.7)	389 (67.4)	465 (68.0)	435 (11.2)
V_z_/F, L	2290 (50.7)	3870 (22.2)	3580 (62.9)	2640 (62.6)	2260 (68.7)	2880 (15.1)

^*a*^ Except where indicated, data are geometric mean (coefficient of variation, %). Data include participants from study VNRX-7145-101.

^*b*^ AUCinf, area under the concentration-time curve through infinity; CL/F, apparent total clearance; Cmax, maximum concentration; Tmax, time of maximum concentration; Vz/F, volume of distribution in the terminal phase.

^*c*^ Data are median hours (range).

Following single doses of LED-E 100 to 1,000 mg, the AUC_inf_ of LED-E was ≤2% of LED exposures, suggesting extensive presystemic conversion of the prodrug to the active drug. Exposure (as measured by AUC) increased in a dose-proportional manner across the dose range, from a geometric mean AUC_inf_ of 0.177 h·µg/mL (LED-E 100 mg) to 2.300 h·µg/mL (LED-E 1000 mg). Geometric mean plasma concentrations increased rapidly following single doses of LED-E with maximum plasma concentrations (*C*_max_) reached 0.5 to 0.75 h post-dose (Table 2). The geometric mean *t*_1/2_ was 2.8 to 4.7 h, with no clear trend as dose increased.

Following coadministration with ceftibuten, an absence of an interaction for AUC_inf_ was observed for LED (GMR, 0.99 [90% CI, 0.95 to 1.03]) ; LED-E AUC_inf_ (GMR, 0.94 [0.85 to 1.04]); and cis-CTB (GMR, 0.88 [0.81 to 0.95]). For C_max_, an absence of interaction was demonstrated for LED (GMR, 0.96 [90% CI, 0.89 to 1.03]). For trans-CTB AUC_inf_ (GMR, 0.86 [90% CI, 0.79 to 0.93]), LED-E *C*_max_ (GMR, 0.76 [90% CI, 0.66 to 0.87]), Cis-CTB *C*_max_ (GMR, 0.88 [0.79 to 0.97]), and trans-CTB *C*_max_ (GMR, 0.85 [90% CI, 0.77 to 0.94]), an interaction cannot be ruled out (Table 3).

**TABLE 3:** Summary of statistical analysis of potential drug-drug interactions following a single dose of ceftibuten (CTB) 400 mg + ledaborbactam etzadroxil (LED-E) 500 mg^*a*^

Analyte	Pharmacokinetic parameter (unit)^*c*^^,*d*^	Geometric LS means^*b*^		Ratio of test/Reference		
		Test	Reference	Estimate	90% CI^*e*^	
					Lower	Upper
Ledaborbactam^*f*^	*C*_max_, µg/mL	13.16	13.74	0.96	0.89	1.03
	AUC_inf_, h·µg/mL	65.36	66.30	0.99	0.95	1.03
Ledaborbactam etzadroxil^*f*^	*C*_max_, µg/mL	0.787	1.037	0.76	0.66	0.87
	AUC_inf_, h·µg/mL	1.068	1.138	0.94	0.85	1.04
Cis-ceftibuten^*g*^	*C*_max_, µg/mL	15.41	17.56	0.88	0.79	0.97
	AUC_inf_, h·µg/mL	79.03	90.09	0.88	0.81	0.95
Trans-ceftibuten^*g*^	*C*_max_, µg/mL	1.319	1.552	0.85	0.77	0.94
	AUC_inf_, h·µg/mL	8.946	10.40	0.86	0.79	0.93

^*a*^ Data are from study VNRX-7145-102.

^*b*^ LS, least-squares.

^*c*^ Units are for geometric LS means, and do not apply to the ratios or corresponding 90% CIs.

^*d*^ AUCinf, area under the concentration-time curve from time zero to infinity; Cmax, maximum concentration.

^*e*^ CI, confidence interval.

^*f*^ For ledaborbactam and ledaborbactam etzadroxil, test product was a single dose of CTB 400 mg + LED-E 500 mg; reference product was a single dose of LED-E 500 mg. The analysis of variance model included fixed effects for treatment, period, and sequence and a random effect for participant within sequence. An absence of interaction was indicated by a 90% CI contained within an equivalence margin of 0.80 to 1.25.

^*g*^ For ceftibuten, test product was a single dose of CTB 400 mg + LED-E 500 mg; reference product was CTB 400 mg. The analysis of variance model included fixed effects for treatment, period, and sequence and a random effect for participant within sequence. An absence of interaction was indicated by a 90% CI contained within an equivalence margin of 0.80 to 1.25.

##### Urine results

Urine samples were analyzed following single doses of LED-E 300 to 1,000 mg (Table S2). For concentrations of LED and LED-E, the total amount excreted during the 48-h post-dose period (Ae_0-48_) increased with increasing dose, from a geometric mean of 131 to 515 mg for LED. Minimal amounts of LED-E (0.006 to 0.05 mg) were excreted in urine. The fraction of total derived material (LED-E +LED equivalent) excreted during the 48-h post-dose period ranged from 65% to 81% and was comprised almost entirely of unchanged LED. Renal clearance (CLr) of LED was similar across dose groups.

### Multiple doses of LED-E

#### Plasma results

Pharmacokinetic parameters were assessed following 10 days of dosing with LED-E 75 to 500 mg q8h. Arithmetic mean plasma concentration-time profiles for LED on Day 10 are shown in Fig. 1B. On Day 10, LED concentrations increased rapidly after administration, reaching *C*_max_ at 0.75 to 1.50 h post-dose (Table 4). LED exposure increased less than dose proportionally, with geometric mean AUC_tau_ (0 to 8 h post-dose) of 13.900 h·µg/mL (LED-E 75 mg q8h) to 69.988 h·µg/mL (LED-E 500 mg). For all dose levels assessed, AUC_tau_ and *C*_max_ increased approximately 30% to 35% from the first to last dose. Day 10 *t*_1/2_ was 9.3 to 12.5 h across dose groups. Steady-state LED plasma concentrations appeared to be reached by Day 3.

**TABLE 4:** Plasma pharmacokinetic parameters for ledaborbactam and ledaborbactam etzadroxil on dosing Day 10 following multiple doses of ledaborbactam etzadroxil (LED-E)^*a*^

Parameter^*b*^	LED-E 75 mg q8h^*c*^ (*n* = 9)	LED-E 150 mg q8h^*c*^ (*n* = 8)	LED-E 300 mg q8h^*c*^ (*n* = 8)	LED-E 500 mg q8h^*c*^ (*n* = 9)
Ledaborbactam				
*T*_max_, h^*d*^	1.00 (0.50–1.75)	0.75 (0.75–1.75)	1.13 (0.75–1.75)	1.50 (0.75–3.00)
*C*_max_, µg/mL	3.690 (25.5)	5.870 (11.4)	11.600 (31.9)	15.788 (29.9)
AUC_tau_, h·µg/mL	13.900 (16.8)	20.800 (15.6)	40.900 (13.7)	69.988 (26.4)
*t*_1/2_, h	9.3 (8.5)	11.4 (25.0)	11.3 (10.4)	12.5 (6.5)
Rac (AUC_tau_)	1.32 (15.8)	1.21 (6.96)	1.30 (7.54)	1.35 (16.7)
Ledaborbactam etzadroxil				
*T*_max_, h^*d*^	0.50 (0.50–0.75)	0.50 (0.25–0.75)	0.50 (0.25–0.75)	0.75 (0.50–1.50)
*C*_max_, µg/mL	0.122 (121)	0.447 (100)	0.629 (103)	0.919 (51.6)
AUC_tau_, h·µg/mL	0.0855 (88.1)	0.309 (72.9)	0.480 (61.7)	1.048 (37.0)
*t*_1/2_, h	3.07 (23.8)	4.78 (23.4)	6.75 (18.5)	9.14 (28.1)
Rac (AUC_tau_)	1.08 (39.7)	1.20 (37.4)	0.92 (41.1)	1.07 (30.4)
CL_SS_/F, L/h	877 (88.1)	486 (72.9)	625 (61.7)	477 (37.0)
V_z_/F, L	3880 (70.7)	3350 (54.1)	6080 (65.0)	6289 (31.0)

^*a*^ Except where indicated, data are geometric mean (coefficient of variation, %). Data include participants from studies VNRX-7145-101 and VNRX-7145-102.

^*b*^ AUCtau, area under the concentration-time curve through the last quantifiable concentration; CLSS/F, apparent total clearance at steady state after oral administration; Cmax, maximum concentration; Rac, accumulation ratio; Tmax, time of maximum concentration; Vz/F, volume of distribution in the terminal phase.

^*c*^ q8h, every 8 h.

^*d*^ Data are median hours (range).

Plasma concentrations of LED-E also increased rapidly following multiple doses of LED-E 75 to 500 mg q8h. Exposure increased with increasing dose, from a geometric mean AUC_tau_ of 0.0855 h·µg/mL (LED-E 75 mg q8h) to 1.048 h·µg/mL (LED-E 500 mg q8h). Geometric mean *C*_max_ reached 0.5 to 0.75 h post-dose on Day 10 and increased from 0.122 µg/mL (LED-E 75 mg q8h) to 0.919 µg/mL (LED-E 500 mg q8h). The geometric mean *t*_1/2_ increased with dose, from 3.07 h (LED-E 75 mg q8h) to 9.14 h (LED-E 500 mg q8h).

On Day 10 following multiple doses of CTB + LED, the geometric mean LED AUC_tau_ increased 24%–26% from the first to the last dose (Table S3). In both dose groups, the LED-E geometric mean AUC_tau_ was unchanged from the first to last dose. The geometric mean AUC_tau_ for cis-CTB increased 44% to 52% from the first to last dose. The geometric mean AUC_tau_ for trans-CTB increased 64% to 75% from the first to the last dose.

#### Urine results

Ae_0–48_ for LED increased with increasing dose, from a geometric mean of 51.6 mg (LED-E 75 mg q8h) to 345 mg (LED-E 500 q8h), and from below the limit of quantification (LED-E 75 mg q8h) to 0.03 mg (LED-E 500 mg q8h) for LED-E (Table S2). Renal CLr of LED increased with increasing dose; renal CLr of LED-E was low and generally similar across dose groups. At steady state in the LED-E 500 mg q8h cohort, urinary excretion of LED-E-derived material (i.e., unchanged LED) was 84.4% over the 8-h dosing interval.

On Day 10, for both the CTB 400 mg +LED E 300 mg q8h and CTB 400 mg +LED E 500 mg q8h dose groups, the geometric mean Fe_0-8_ of total derived material (LED-E +LED equivalent) was 72% to 77% of the last dose, with low levels of unchanged LED-E excretion. The geometric mean Fe_0-8_ of LED-E recovered in urine was less than 0.01% of the last dose (Table S4). The mean urinary Fe_0-8_ of cis-CTB was 50% to 68% of the last dose, and the mean Fe_0-8_ of trans-CTB was 20% to 23% of the last dose.

## DISCUSSION

LED-E, an oral prodrug of the active β-lactamase inhibitor LED, is being developed as part of an oral combination with CTB, a third-generation cephalosporin, to address the need for effective oral options to treat complicated urinary tract infections (cUTIs) and pyelonephritis caused by drug-resistant Enterobacterales (5–9). CTB + LED has demonstrated potent *in vitro* activity against drug-resistant Enterobacterales, including strains producing ESBLs, AmpC, and serine carbapenemases such as KPC and OXA-48. LED at a fixed concentration of 4  µg/mL restores CTB susceptibility, reducing MIC values by 32- to 1,024-fold, depending on the β-lactamase type, with potentiated MIC_90_ values ranging from ≤0.25 to ≤2 µg/mL (7, 8). *In vitro* studies have demonstrated that more than 98% of ESBL-positive isolates were inhibited at MICs ≤ 1 µg/mL, and the combination showed significant activity against KPC (85.9%) and OXA-48 (82.9%) producers (7). At ceftibuten breakpoints of ≤1 mg/L (EUCAST), 92.5% of 200 carbapenem-resistant *K. pneumoniae* isolates were inhibited by ceftibuten-ledaborbactam compared with only 4.5% for ceftibuten alone (10).

LED-E was well tolerated at all dose levels evaluated when dosed alone or in combination with CTB, with no serious or severe TEAEs following single or multiple doses. Gastrointestinal symptoms were the most common system organ class TEAEs reported for the active intervention groups. All gastrointestinal symptoms were mild and did not result in treatment discontinuation.

Pharmacokinetic analysis of single doses of LED-E revealed a dose-proportional increase in plasma levels (as defined in the pharmacokinetic analysis section) of LED as the LED-E dose increased from 100 to 1,000 mg. LED exposure increased less than proportional to dose following 10 days of q8h dosing. Steady-state plasma concentrations were reached by Day 3, and AUC_tau_ increased approximately 30% to 35% from the first to last doses, indicating moderate accumulation of LED in plasma after 10 days of dosing. The low plasma exposure of LED-E (<2%) relative to LED exposure confirmed extensive conversion to the active β-lactamase inhibitor. The increase in LED half-life with dose may be an artifact resulting from plasma concentrations falling below the assay LLOQ before reaching the terminal phase at lower doses. Urinary excretion, almost entirely as unchanged LED, is the primary route of elimination following oral dosing of LED-E (84% of the drug was excreted in urine following 500 mg q8h dosing), supporting its potential role in the treatment of cUTI. These results indicate that dose adjustments will be required for both ceftibuten (11, 12) and ledaborbactam etzadroxil with increasing renal impairment and that LED bioavailability is at least 68%.

The amount of unchanged LED excreted into the urine over the dosing interval at steady state (Table S2) following administration of LED-E doses between 300 and 500 mg results in exposures that exceed established plasma stasis targets for LED (13) determined in a murine thigh model or a fixed concentration of 4 µg/mL used in *in-vitro* susceptibility testing and correlated with pharmacodynamic response in a murine thigh model (5). Although a ledaborbactam 1-log_10_ PK-PD target magnitude was not achievable in the murine thigh model, a humanized ceftibuten-ledaborbactam dose reduced colony counts in kidney and bladder by 3–6 log_10_ compared to saline dosed controls in a murine cUTI model (14).

Plasma pharmacokinetics results were similar between cohorts that received multiple doses of the CTB + LED E combination (Table S3) and those that received LED-E alone (Table 4). Co-administration of a single dose of CTB 400 mg with LED-E 500 mg demonstrated a lack of interaction for LED and cis-CTB AUC,_inf_ which are considered to be most closely associated with efficacy. *C*_max_ was the same or lower following coadministration for LED, LED-E, and both CTB isomers, suggesting no additional safety risk with coadministration. These results support the conclusion that there was no clinically meaningful pharmacokinetic interaction following coadministration of LED-E and CTB.

The findings from these 2 Phase 1 studies support the continued development of CTB + LED-E as a carbapenem-sparing therapeutic option for treating cUTIs and other serious infections caused by drug-resistant Enterobacterales. An effective oral combination has the potential to reduce the need for intravenous therapies, hospitalization, and healthcare costs (7, 10). The overall safety profile of the CTB + LED-E combination was consistent with what has been observed with CTB alone (11, 12), and the pharmacokinetic profile of LED is favorable for co-administration with CTB.

## MATERIALS AND METHODS

### Study design

VNRX-7145-101 was a two-part randomized, double-blind, placebo-controlled, sequential-group study of the safety and pharmacokinetics of single ascending doses (SAD, Part 1) and multiple ascending doses (MAD, Part 2) of LED-E in 48 healthy adults. In Part 1, participants in each cohort were randomized in a 3:1 ratio to receive a single dose of LED-E (100, 200, 300, 500, 800, or 1,000 mg) or placebo. In Part 2, participants in each cohort were randomized in a 3:1 ratio to receive multiple doses of LED-E (75, 150, or 300 mg) or placebo q8h for 10 days. Cohort 3 (300 mg q8h) was designed to enroll 12 participants, but enrollment was halted at 11 participants due to slow enrollment at the clinical site.

VNRX-7145-102 was a three-part randomized study of CTB and LED-E in healthy adults. Part 1 used an open-label, cross-over design to evaluate safety and drug-drug interaction (DDI) of a single dose of LED-E 500 mg, CTB 400 mg, and CTB + LED-E combination in 18 participants randomized to a dosing sequence in a 1:1:1:1:1:1 ratio. Part 2 evaluated multiple doses of LED-E 500 mg or placebo q8h, administered for 10 days, in 12 participants randomized in a 3:1 ratio. Part 3 used a double-blind, parallel-group design to evaluate safety and pharmacokinetics of CTB 400 mg + LED-E 300 mg, CTB 400 mg + LED-E 500 mg, or placebo q8h for 10 days, in 24 participants randomized in a 5:5:2 ratio. Part 3 was designed to enroll 24 participants, but enrollment was halted at 23 participants due to slow enrollment at the clinical site.

The study protocols were reviewed and approved by an institutional review board. Both studies were done in accordance with the ethical principles that have their origin in the Declaration of Helsinki, with International Council for Harmonization Guideline for Good Clinical Practice, and with all applicable regulatory requirements. All participants provided written informed consent before any screening procedures were performed. The studies were registered on ClinicalTrials.gov (NCT04243863 and NCT04877379).

### Participants

The studies enrolled healthy men and women ages 18 to 45 years (VNRX-7145-101) or 18 to 55 years (VNRX-7145-102) with body mass index ≥18.5 and ≤32.0 kg/m^2^ and normal vital signs and clinical laboratory values at screening and Day –1. Participants were required to abstain from alcohol, caffeine, and xanthine-containing products beginning ≥2 days before admission to the research site through discharge. Key exclusion criteria were presence of a chronic condition; history of drug allergy including hypersensitivity to cephalosporins, penicillins, or β-lactam antibiotics (VNRX-7145-102 only); positive drug screen; confirmed or suspected COVID-19 infection (VNRX-7145-102 only); or positive screening tests for hepatitis B surface antigen, anti-hepatitis C virus antibodies, or anti-human immunodeficiency virus 1 or 2 antibodies. Participants were required to discontinue any medications, vitamins, supplements, nicotine, and vaping products at least 14 days before Day –1; exceptions were acetaminophen at doses ≤ 2 g/day (VNRX-7145-101) or ≤3 g/day (VNRX-7145-102) and hormonal contraceptives.

### Assessments

In both studies, safety analyses included all participants in both VNRX-7145-101 and VNRX-7145-102 who received at least one dose of study medication. Safety was assessed via ongoing monitoring of adverse events, symptom-directed physical examinations, and changes in clinical laboratory measures, vital signs, and ECGs.

For determination of single-dose and steady-state plasma pharmacokinetics, serial blood samples were collected at the following timepoints in VNRX-7145-101: in the SAD (Part 1), pre-dose and 0.25, 0.5, 0.75, 1, 1.5, 2, 2.5, 3, 4, 5, 6, 8, 12, 16, 24, 36, and 48 h post-dose; in the MAD (Part 2), 0.25, 0.5, 0.75, 1, 1.25, 1.5, 1.75, 2, 2.5, 3, 4, 5, 6, 8, h after the Day 1 dose, 0.25, 0.5, 0.75, 1, 1.25, 1.5, 1.75, 2, 2.5, 3, 4, 5, 6, 8, 12, 16, 24, 36, and 48 h after the Day 10 dose, and pre-dose on Days 1−5 and 10. Serial blood samples were collected at the following timepoints in VNRX-7145-102: in Part 1 (dosing on Days 1, 5, and 9), predose and 0.5, 0.75, 1, 1.25, 1.5, 1.75, 2, 2.5, 3, 4, 5, 6, 8, 12, 16, 24, and 48 h postdose; in Part 2, predose on Days 1-5 and Day 10, and 0.5, 0.75, 1, 1.25, 1.5, 1.75, 2, 2.5, 3, 4, 5, 6, and 8 h after the first dose on Day 1 and the Day 10 dose, and 12, 16, 24, and 48 h after the Day 10 dose; in Part 3, predose and 0.5, 0.75, 1, 1.25, 1.5, 1.75, 2, 2.5, 3, 4, 5, 6, and 8 h after the Day 1 and 10 dose and 12, 16, 24, and 48 h after the Day 10 dose.

In VNRX-7145-101, urine samples for pharmacokinetic analysis were collected predose and at 0 to 4, 4 to 8, 8 to 12, 12 to 24, and 24 to 48 h after single doses and on Day 10 following the last dose. Urine samples were collected in Parts 2 and 3 of VNRX-7145-102 at the following times: predose on Days 1 and 10 and at 0 to 4, 4 to 8, 8 to 12, 12 to 24, and 24 to 48 h after the Day 10 dose.

Plasma and urine pharmacokinetics samples were assayed for LED-E, LED, and CTB, as appropriate using validated bioanalytical methods. Liquid chromatography–tandem mass spectrometry (LC-MS/MS) was used to analyze human acidified sodium fluoride potassium oxalate plasma samples for LED-E and LED using a calibration standard of 0.500 to 500 ng/mL (LED-E) and 10.0 to 10,000 ng/mL (LED) based on the analysis of 0.100 mL of acidified plasma. Quantitation used separate weighted 1 /x^2^ linear least-squares regression analyses generated from calibration standards. Exploratory screening for metabolites was performed for some samples.

### Pharmacokinetic and statistical analyses

For VNRX-7145-101, individual concentration-time data were summarized by cohort using descriptive statistics for all participants who received at least one dose of the study treatment and provided at least one evaluable plasma concentration. Single-dose plasma pharmacokinetic parameters were determined in Part 1, and both single-dose (Day 1) and multiple-dose (Day 12) parameters were determined in Part 2. Calculation of λ_Z_ was based on best fit of concentrations in the observed terminal elimination phase of a profile, and statistical inclusion of any parameter based on λ_Z_ required the adjusted goodness-of-fit statistic (R^2^_adj_) to be 0.8 or higher. Dose proportionality was analyzed using a power model in which pharmacokinetic exposure parameters and dose were converted to the natural log and linear regression results were reported, with the slope as the power estimate (15). If the 90% CI for the slope contained 1, then the hypothesis of dose proportionality for AUC or *C*_max_ could not be rejected. In Part 2, achievement of steady-state concentrations was assessed using Tukey’s multiple comparison test for pre-dose and all dosing days (Days 2 to 10) for each dose level separately.

For VNRX-7145-102, descriptive statistics was used to summarize plasma concentrations by study part and treatment at each assessment time point for all participants who received at least 1 dose of study drug and had at least one evaluable post-dose concentration measurement. Plasma pharmacokinetic parameters were calculated using noncompartmental analysis of the plasma and urine concentration-time data. In Part 1, the effect of coadministration of CTB + LED-E on LED-E, LED, cis-CTB, and trans-CTB plasma pharmacokinetic parameters was assessed. AUC_inf_ and *C*_max_ were natural log-transformed before analysis, using a linear mixed effects model. The analysis of variance (ANOVA) model included fixed effects for treatment, period, and sequence and a random effect for participant within sequence. Point estimates, geometric means, geometric mean ratios, and corresponding 90% CIs were calculated. An absence of interaction was indicated by a 90% CI that was contained within 0.80 to 1.25.

In both studies, plasma pharmacokinetic parameters were calculated using WinNonlin (Certara; Radnor, PA), and urine pharmacokinetic parameters were calculated using SAS (SAS Institute; Cary, NC).

## SUPPLEMENTAL MATERIAL

Supplemental tables aac.00210-25-s0001.docx Tables S1 to S5.

ASM does not own the copyrights to Supplemental Material that may be linked to, or accessed through, an article. The authors have granted ASM a non-exclusive, world-wide license to publish the Supplemental Material files. Please contact the corresponding author directly for reuse.
