# Supplementary material for: Safety and pharmacokinetics of single and multiple doses of ledaborbactam etzadroxil with or without ceftibuten in healthy volunteers
Source: Antimicrob Agents Chemother. 2025 Aug 5;69(9):e00210-25. doi: 10.1128/aac.00210-25 (PMC12406652; doi:10.1128/aac.00210-25)
Supplement: Supplemental tables — Tables S1 to S5. [file aac.00210-25-s0001.docx]

## Supplemental Material

**Supplemental Table 1.** Demographics and baseline characteristics

| **Characteristic^a^** | LED-E ± CTB^b^ (n = 109) | Placebo (n = 27) | Total (N= 136) |
| --- | --- | --- | --- |
| Age, years, mean (SD) | 30.7 (8.5) | 32.4 (8.4) | 31.0 (8.5) |
| Male | 48 (44.0) | 11 (40.7) | 59 (43.4) |
| Race |  |  |  |
| White | 80 (73.4) | 16 (59.3) | 96 (70.6) |
| Black or African American | 15 (13.8) | 9 (33.3) | 24 (17.6) |
| Multiple races | 9 (8.3) | 2 (7.4) | 11 (8.1) |
| Other^c^ | 5 (4.6) | 0 | 5 (3.7) |
| Not Hispanic or Latino | 64 (58.7) | 17 (63.0) | 81 (59.6) |
| BMI, kg/m^2^, mean (SD) | 25.4 (3.4) | 26.1 (2.6) | 25.5 (3.2) |

Except where otherwise specified, data are n (%). Data include participants from studies VNRX-7145-101 and VNRX-7145-102.

^a^BMI, body mass index; SD, standard deviation.

^b^CTB, ceftibuten; LED-E, ledaborbactam etzadroxil.

^c^Participants categorized as “other” self-identified as American Indian or Alaska Native (n = 2), Asian (n = 2), or Native Hawaiian or other Pacific Islander (n = 1).

**Supplemental Table 2.** Urinary pharmacokinetic parameters following single and multiple doses of ledaborbactam etzadroxil (LED-E).

| **Parameter^a^** | LED-E Single Doses^b^ | | | | LED-E Multiple Doses q8h^c^ | | | |
| --- | --- | --- | --- | --- | --- | --- | --- | --- |
|  | 300 mg (n=6) | 500 mg (n=6) | 800 mg (n=6) | 1000 mg (n=6) | 75 mg^d^ (n=9) | 150 mg (n=8) | 300 mg (n=8) | 500 mg (n=9) |
| *Ledaborbactam* | | | | | | | | |
| Ae_0–8_, mg | 113 (23.3) | 190 (14.1) | 364 (6.14) | 459 (16.6) | 43.1 (6.95) | 73.1 (10.0) | 137 (34.2) | 283 (7.8) |
| Ae_0–48_, mg | 131 (20.3) | 220 (12.2) | 438 (3.3) | 515 (16.9) | 51.6 (7.80) | 86.6 (11.1) | 166 (27.8) | 345 (11.2) |
| CLr, L/h | 3.98 (23.6) | 3.20 (17.9) | 3.52 (24.4) | 4.53 (13.2) | 2.65 (13.6) | 3.11 (13.4) | 3.09 (39.0) | 4.92 (19.9) |
| *Ledaborbactam etzadroxil* | | | | | | | | |
| Ae_0–8_, mg | 0.00779 (51.0) | 0.0212 (38.4) | 0.0301 (49.2) | 0.0542 (68.6) | — | — | — | 0.0273 (38.5) |
| Ae_0–48_, mg | 0.00573 (106) | 0.0212 (38.4) | 0.0301 (49.2) | 0.0542 (68.6) | — | 0.00488 (47.9) | 0.00378 (218) | 0.0273 (38.5) |
| CLr, L/h | 0.0132 (109) | 0.0165 (53.9) | 0.0175 (38.4) | 0.0236 (62.5) | — | 0.0148 (60.4) | 0.00728 (311) | 0.0260 (32.6) |
| *Total derived material, ledaborbactam etzadroxil* | | | | | | | | |
| Fe_0–8_, %^e^ | 56.1 (23.3) | 56.4 (14.1) | 67.7 (6.1) | 68.3 (16.6) | 85.4 (7.0) | 72.5 (10.0) | 68.1 (34.2) | 84.2 (7.8) |
| Fe_0–48_, %^e^ | 64.9 (20.3) | 65.5 (12.2) | 81.4 (3.3) | 76.5 (16.9) | — | — | — | — |

Data are geometric mean (geometric coefficient of variation, %). Data are from studies VNRX-7145-101 and VNRX-7145-102.

^a^Ae_0–8_, amount of drug recovered in urine from time 0 through 8 hours post-dose; Ae_0-48_, amount of drug recovered in urine from time 0 through 48 hours post-dose; CLr, renal clearance; Fe_0-8_, fraction of drug recovered in urine from time 0 through 8 hours post-dose; Fe_0-48_, fraction of drug recovered in urine from time 0 through 48 hours post-dose.

^b^Urine pharmacokinetics were not performed for 100 mg and 200 mg dose levels.

^c^q8h, every 8 hours.

^d^Ae_0-8_ and Ae_0-48_ were not calculated at 75 mg q8h for ledaborbactam etzadroxil.

^e^Fe parameters were based on LED-E–total derived material, ie, LED-E + LED equivalent, where LED equivalent was calculated as LED Ae × (LED-E molecular weight / LED molecular weight). Molecular weights are 391.23 (LED-E) and 263.10 (LED).

**Supplemental** **Table 3.** Plasma pharmacokinetic parameters on Day 10, following multiple doses of ceftibuten (CTB) + ledaborbactam etzadroxil (LED-E).

| **Parameters^a^** | CTB 400 mg + LED-E 300 mg^b^ (n=10) | CTB 400 mg + LED-E 500 mg q8h^b^ (n=10) |
| --- | --- | --- |
| *Ledaborbactam* |  |  |
| *T*_max_, h^c^ | 1.00 (0.75–2.00) | 1.50 (0.50–3.00) |
| *C*_max_, µg/mL | 8.469 (35.3) | 16.152 (25.0) |
| AUC_tau_, h•µg/mL | 33.319 (25.3) | 62.239 (16.8) |
| *t*_1/2_, h | 11.4 (10.6) | 12.3 (8.4) |
| Rac (AUC_tau_) | 1.24 (10.4) | 1.26 (14.0) |
| *Ledaborbactam etzadroxil* |  |  |
| *T*_max_, h^c^ | 0.5 (0.50–1.75) | 0.51 (0.50–1.00) |
| *C*_max_, µg/mL | 0.467 (56.5) | 1.175 (60.4) |
| AUC_tau_, h•µg/mL | 0.463 (34.1) | 1.090 (28.7) |
| *t*_1/2_, h | 7.68 (29.6) | 10.7 (23.4) |
| Rac (AUC_tau_) | 1.03 (33.4) | 0.96 (22.4) |
| *Cis-CTB* |  |  |
| *T*_max_, h^c^ | 2.25 (1.75–4.00) | 2.50 (1.25–4.00) |
| *C*_max_, µg/mL | 21.803 (10.9) | 23.801 (22.8) |
| AUC_tau_, h•µg/mL | 92.982 (10.6) | 104.817 (21.3) |
| *t*_1/2_, h | 2.78 (11.0) | 2.61 (21.2) |
| Rac (AUC_tau_) | 1.44 (18.7) | 1.52 (18.3) |
| *Trans-CTB* |  |  |
| *T*_max_, h^c^ | 3.00 (1.75–4.00) | 3.00 (2.00–5.02) |
| *C*_max_, µg/mL | 1.621 (9.2) | 1.912 (27.3) |
| AUC_tau_, h•µg/mL | 8.734 (13.8) | 10.138 (22.1) |
| *t*_1/2_, h | 3.71 (11.0) | 3.84 (27.5) |
| Rac (AUC_tau_) | 1.64 (23.4) | 1.75 (30.4) |

Except where indicated, data are geometric mean (coefficient of variation, %). Data are from study VNRX-7145-102.

^a^AUC_tau_, area under the concentration-time curve through the last quantifiable concentration; cis-CTB, cis-ceftibuten; *C*_max_, maximum concentration; Rac, accumulation ratio; *t*_1/2_, observed terminal half-life; *T*_max_, time of maximum concentration; trans-CTB, trans-ceftibuten.

^b^CTB, ceftibuten; LED-E, ledaborbactam etzadroxil.

^c^Data are median (range).

**Supplemental Table 4.** Cumulative recovery of ledaborbactam etzadroxil, ledaborbactam, cis-ceftibuten, and trans-ceftibuten in urine on Day 10 following multiple doses of study drug.

| **Analyte^a^** | Pharmacokinetic Parameter^b^ | | |
| --- | --- | --- | --- |
|  | Ae_0–8_, mg | Fe_0–8_, % | CLr, L/h |
| *Ledaborbactam^c^* |  |  |  |
| CTB 400 mg + LED-E 300 mg q8h (n=9) | 156 (11.3) | — | 5.15 (13.4) |
| CTB 400 mg + LED-E 500 mg q8h (n=10) | 243 (22.8)^d^ | — | 4.49 (26.4) |
| *Ledaborbactam etzadroxil^c^* |  |  |  |
| CTB 400 mg + LED-E 300 mg q8h (n=9) | 0.0113 (41.4) | 0.00377 (41.4) | 0.0241 (42.0) |
| CTB 400 mg + LED-E 500 mg q8h (n=10) | 0.0271 (58.4)^d^ | 0.00542 (58.4)^d^ | 0.0252 (42.8) |
| *Total derived material, ledaborbactam etzadroxil^c^* |  |  |  |
| CTB 400 mg + LED-E 300 mg q8h (n=9) | 156 (11.3) | 77.4 (11.3) | — |
| CTB 400 mg + LED-E 500 mg q8h (n=10) | 244 (22.8)^d^ | 72.4 (22.8)^d^ | — |
| *Cis-ceftibuten* |  |  |  |
| CTB 400 mg + LED-E 300 mg q8h (n=9) | 270 (11.9) | 67.6 (11.9) | 3.24 (11.3) |
| CTB 400 mg + LED-E 500 mg q8h (n=10) | 202 (32.7)^d^ | 50.5 (32.7)^d^ | 2.21 (23.2) |
| *Trans-ceftibuten* |  |  |  |
| CTB 400 mg + LED-E 300 mg q8h (n=9) | 81.9 (29.3) | 20.5 (29.3) | 12.3 (28.5) |
| CTB 400 mg + LED-E 500 mg q8h (n=10) | 90.9 (33.4)^d^ | 22.7 (33.4)^d^ | 11.2 (32.5) |

Data are geometric mean (geometric coefficient of variation, %), and are from study VNRX-7145-102.

^a^CTB, ceftibuten; LED-E, ledaborbactam etzadroxil; q8h, every 8 hours.

^b^Ae_0-8_, amount of drug recovered in urine from time 0 through 8 hours post-dose; CLr, renal clearance; Fe_0-8_, fraction excreted in urine from time 0 through 8 hours post-dose.

^c^Ae_0-8_ and Fe_0-8_ calculations for LED-E–total derived material, ie, LED-E + LED equivalent, where LED equivalent was calculated as LED Ae × (LED-E molecular weight / LED molecular weight). Molecular weights are 391.23 (LED-E) and 263.10 (LED).

^d^n=9 for Ae_0-8_ and Fe_0-8_ calculations

**Supplemental Table 5.** Summary of bioanalytical assay characteristics

| Analyte^a^ | LED-E | | LED | |
| --- | --- | --- | --- | --- |
| Matrix | Plasma^c^ | Urine^d^ | Plasma^c^ | Urine^d^ |
| Platform^b^ | LC-MS/MS equipped with HPLC column | LC-MS/MS equipped with HPLC column | LC-MS/MS equipped with HPLC column | LC-MS/MS equipped with HPLC column |
| Calibration Range | 0.0005 to 0.5 µg/mL | 0.001 to 1 ug/mL | 0.01 to 1 µg/mL | 0.1 to 100 µg/mL |
| Standard Calibration Curve Performance |  |  |  |  |
| Cumulative accuracy (%bias) | -3.1% to 3.0% | -2.5% to 2.0% | -2.3% to 1.2% | -2.9% to 3.6% |
| Cumulative Precision (%CV) | ≤ 5.8% | ≤ 3.3% | ≤ 4.7% | ≤ 4.3% |
|  |  |  |  |  |
| QC Performance |  |  |  |  |
| Cumulative Accuracy (%bias) | -2.8% to 1.2% | -0.6% to 7.0% | -9.1% to -2.4% | -1.6% to 8.7% |
| Interbatch %CV | ≤ 7.4% | ≤ 7.2% | ≤ 9.1% | ≤ 10.8% |

^a^LED-E, ledaborbactam etzadroxil; LED, ledaborbactam.

^b^LC-MS/MS, liquid chromatography – tandem mass spectrometry; HPLC, high performance liquid chromatography.

^c^LC-MS/MS (Sciex API 4000) equipped with HPLC column (Kinetix 2.6 µM XB-C18, 2.1x50 mm)

^d^LC-MS/MS (Sciex API 4000) equipped with HPLC column (Waters Xbridge C18 2.1x50 mm, 2.5µm)
